# Gold oxide formation on Au(111) under CO oxidation conditions at room temperature†

**DOI:** 10.1039/d4cp00611a

**Published:** 2024-08-29

**Authors:** Sabine Wenzel, Dajo Boden, Irene M. N. Groot

**Affiliations:** a Leiden Institute of Chemistry Einsteinweg 55 2333 CC Leiden The Netherlands

## Abstract

Although gold-based catalysts are promising candidates for selective low-temperature CO oxidation, the reaction mechanism is not fully understood. On a Au(111) model catalyst, we observe the formation of gold oxide islands under exposure to atmospheric pressures of oxygen or CO oxidation reaction conditions in an *in situ* scanning tunneling microscope. The gold oxide formation is interpreted in line with the water-enabled dissociation of O_2_ on the step edges of Au(111). Contaminants on the gold surface can strongly promote the gold oxide formation even on the terraces. On the other hand, TiO_2_ nanoparticles on the Au(111) do not show any influence on the formation of the gold oxide and are thus not providing a significant amount of atomic oxygen to the gold at room temperature. Overall, the presence of gold oxide is likely under industrial conditions.

## Abstract 2

On a Au(111) model catalyst, we observe the formation of gold oxide islands under exposure to atmospheric pressures of oxygen or CO oxidation reaction conditions in an *in situ* scanning tunneling microscope.

## Graphical abstract

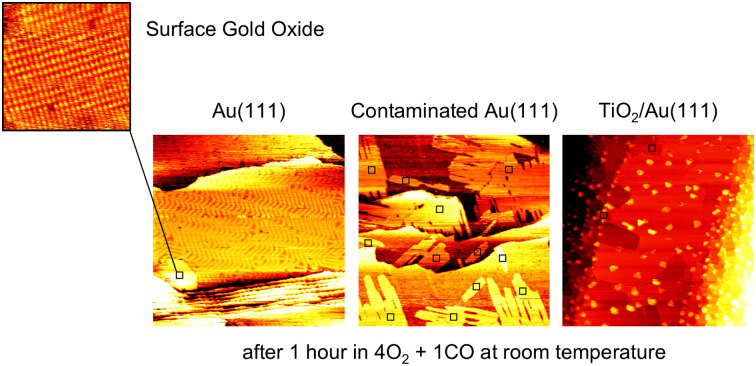

## Introduction

Whereas conventional power plants can be turned on and off as needed, the energy output of wind mills and solar panels varies with the weather and the time of day. Therefore, the transition to sustainable energy sources requires the development of efficient energy-storage methods. A viable option is to store the energy in the form of chemicals such as methanol. The energy stored in the form of methanol can be harvested by converting it to hydrogen *via* methanol steam reforming.^1,2^ However, for the use in fuel cells this hydrogen needs to be free from carbon monoxide, a byproduct of methanol steam reforming, to prevent poisoning of the fuel-cell anode.^3^ For this the traces of CO need to be removed over a second catalyst *via* preferential CO oxidation, which takes place in the hydrogen environment without oxidation of the H_2_:

1 2CO + O_2_ → 2CO_2_

2 2H_2_ + O_2_ ↛ 2H_2_O

Compared to conventional CO oxidation catalysts such as platinum and palladium, gold-based catalysts are more suited for this application, since they are more selective for the oxidation of CO instead of H_2_ at low temperatures.^4–6^ Gold-based catalysts have shown CO oxidation activity at temperatures as low as room temperature.^7^ They are thus additionally interesting for improving the 3-way car catalyst, which is currently not efficiently oxidizing CO during the cold start-up of the car.^8^

There has been research into the catalytic activity of gold nanoparticles deposited on oxide supports for more than 30 years.^9–14^ Inverse model catalysts of oxide particles on metal single crystals are widely applied as well due to the ease of controlled preparation and application of surface-science techniques, especially in ultra-high vacuum.^14–16^ On Au(111) single crystals various oxide nanoparticles have been prepared. Among these are CeO_2_,^17^ MoO_3_,^18^ MgO,^19^ CoO,^20^ Fe_2_O_3_,^21^ and TiO_2_.^22^ However, inverse model catalysts on gold single crystals are not only a useful research tool but can also show even higher CO oxidation activities than their non-inverse counterparts as suggested by Palomino *et al.*^23^ In their study, TiO_2_/Au(111) showed the highest activity of all tested catalysts.

For inverted as well as non-inverted catalysts it is clear that the active site lies in the interface region between support and particles^24–26^ and metal–support interactions have been observed.^23,27,28^ However, the exact oxidation state of gold during the reaction remains under debate. The oxidation of gold is believed to not be possible from molecular oxygen^29–31^ and more specifically under CO oxidation conditions.^32^ There is evidence that a gold oxide prepared with ozone would be less active than metallic gold.^33^ However, other work has suggested that oxidized gold is the active species during CO oxidation.^34,35^ Additionally, there is evidence that water can promote the CO oxidation reaction^36–38^ and could even make the oxide support unnecessary.^39^

Investigating the presence of a gold oxide with spectroscopy techniques is complicated due to the low signals stemming from small amounts of surface oxides as well as the sensitivity of the surfaces to beam damage or even beam-induced oxidation.^30^ Additionally, the sample needs to be exposed to air and thus water between the reaction and the characterization in many laboratories. However, crystalline surface oxides can be detected *via* atomically resolved microscopy as well.^40–43^

Our *in situ* scanning tunneling microscope (STM) setup allows for the imaging of the support and the inverse model catalyst before, during, and after exposure to gases, as well as a rough spectroscopic characterization, without exposure to air in between any of the steps. Here we present evidence for the presence of a gold oxide on Au(111) after exposure to oxygen or CO oxidation conditions at room temperature. We discuss the role of the water background in the reactor, contaminants on the gold substrate, and TiO_2_ nanoparticles.

## Materials and methods

### Surface preparation

The Au(111) single crystal was purchased from SPL and prepared with cycles of argon ion sputtering at 1 kV to 1.4 kV acceleration voltage and annealing to between 800 K and 850 K in UHV. Whenever carbon particles were visible in the STM, the crystal was additionally annealed in 1 × 10^−6^ mbar of O_2_ to between 750 K and 800 K until the carbon was removed. This was followed by at least ten cycles without annealing in oxygen before running an experiment.

For the preparation of the TiO_2_/Au(111) model catalyst titanium was deposited onto the clean, metallic Au(111) using an e-beam evaporator from Oxford applied research. The deposition was performed in an oxygen background of 1 × 10^−6^ mbar at room temperature. Subsequently the crystal was annealed to 850 K in 5 × 10^−6^ mbar of O_2_ for 20 min and cooled down in the same oxygen pressure until below 500 K. To exclude any influence from titanium residue or alloying of titanium with gold the experiments on Au(111) were performed on a crystal that had not been exposed to titanium whereas another crystal was used solely for the experiments on TiO_2_/Au(111).

### Ambient-pressure STM

As described in more detail in ref. 44, the setup allows for scanning tunneling microscopy in ultra-high vacuum as well as in up to 6 bar of gases and at up to 600 K surface temperature. A cut platinum iridium wire (Pt90/Ir10, 0.25 mm) is used as the STM tip. All given bias voltages refer to the voltage applied to the sample and images are taken in constant-current mode. The images were processed in WSxM.^45^ Without leaving the vacuum chamber the sample can be moved between preparation, STM, a low-energy electron diffraction (LEED) setup (Omicron SpectaLEED with NG LEED S control unit), and X-ray photoelectron spectroscopy (XPS).

### Gases

In the following we have used Ar 5.0 from Westfalen with 99.999% purity,^46^ O_2_ 5.0 from Westfalen with 99.999% purity,^47^ and CO 4.7 from air liquide with 99.997% purity.^48^ The water background in the reactor, which is mainly caused by the design of the gas delivery system and likely similar for all gases used, was measured by mass spectrometry as explained in detail previously.^49^ When the reactor was filled to 1 bar Ar or 1 bar O_2_ an order of 1 mbar water was detected.

### X-ray photoelectron spectroscopy

Au 4f and O 1s XP spectra were measured in UHV at room temperature using the aluminium K_α_ line of a VG Microtech dual anode X-ray source and a Clam 2 electron analyzer. For a detailed analysis, the energy axis of all spectra has been calibrated using the Au 4f_3/2_ peak at 84 eV and their intensity has been scaled with respect to the surface area under the gold peak after subtraction of a Shirley background. For comparability the same linear background from 527.5 eV to 533 eV is used for all three oxygen spectra. The area above this background is used for a rough estimation of the oxygen coverage *x*. For this we describe the measured intensity *I*_Au_ of the Au peak and the intensity *I*_O_ of the oxygen peak as:

3 *I*_Au_ = *k*_Au_*N*_Au_*λ*_Au_*Ω*_Au 4f_((1 − *x*) +*x*·*e*^−*d*_O_/*λ*_O_^)

4 *I*_O_ = *x*·*k*_O_*N*_O_*λ*_O_*Ω*_O 1s_(1 − *e*^−*d*_O_/*λ*_O_^)

with the number of atoms *N*_Au_ and *N*_O_ of both species per area and unknown geometric and efficiency factors *k*_Au_ ≈ *k*_O_ of the instrument. Solving the ratio *I*_O_/*I*_Au_ for the coverage *x* leads to5
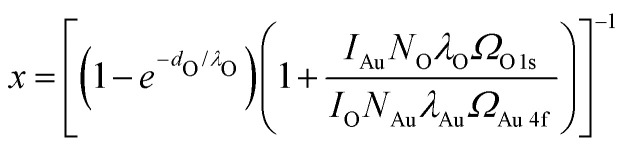


For a detailed derivation of these equations see ref. 50. The cross sections for photoemission at the photon energy of 1486 eV are determined to *Ω*_Au 4f_ = 0.25 Mbarn and *Ω*_O 1s_ = 0.04 Mbarn using ref. 51. For simplicity we estimate the inelastic mean free paths *λ*_Au_ and *λ*_O_ for both species as the one for an electron kinetic energy of 1400 eV travelling through metallic gold *λ* = 1.78 nm (determined with the NIST Database 71 Version 2.1^52^). This value does not have a significant influence on the result however. Based on similar oxide structures observed on Pt(111),^53,54^ we assume one oxygen atom per surface gold atom (*N*_Au_/*N*_O_ = 1). Note that this choice has a significant influence on the resulting coverage, which increases roughly linearly with *N*_Au_/*N*_O_. Finally, the height of the oxide of *d*_O_ = 0.1 nm as seen in the STM is used.

## Results and discussion

### Gold oxide on Au(111)

**Fig. 1:**
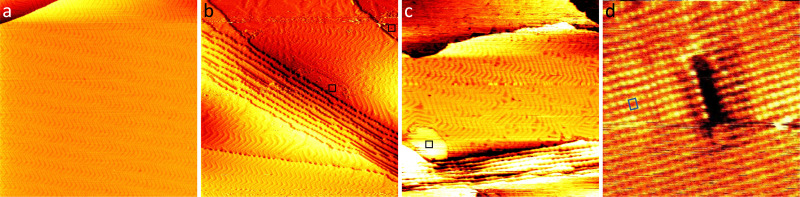
300 nm × 300 nm STM images of (a) the as-prepared Au(111) and the same surface after 1 h in (b) 0.8 bar O_2_ and (c) 1 bar of 4O_2_ + 1CO, respectively. For better visibility of the structure next to the step edges (marked with black squares) the image in (b) is merged with its derivative in a ratio of 1 : 15. (d) 10 nm × 10 nm STM image of the newly formed structure indicating the rectangular unit cell in blue. In the center of the image, a defect in the structure appears black. All images are taken in UHV at room temperature with (a)–(c) −1 V and (d) −0.2 V, and 50 pA.

**Fig. 2:**
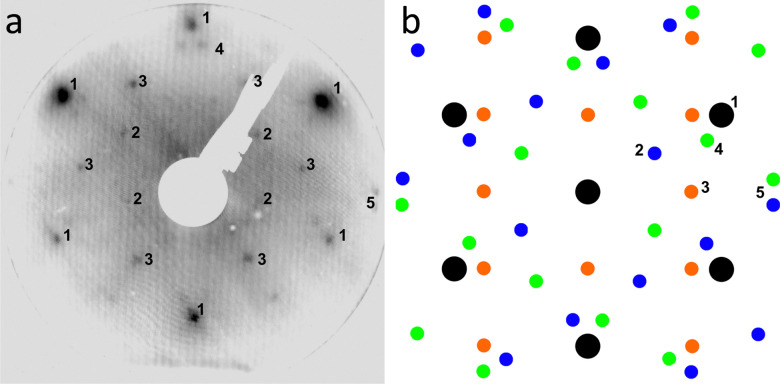
(a) LEED image taken of Au(111) after O_2_ exposure (78 eV electron energy). (b) Simulated LEED image based on the unit cell measured in STM including the Au(111) substrate in black and the three different possible orientations of the oxide overlayer in blue, orange, and green. Corresponding spots in (a) and (b) are marked with numbers 1 to 5. The simulated LEED image is made using LEEDpat.^58^

Fig. 1(a) shows a large-scale STM image of the as-prepared Au(111) surface with the typical herringbone reconstruction characterized by parallel brighter lines with straight sections interrupted regularly by so-called elbows.^55–57^ After exposing this surface to 0.8 bar O_2_ or 1 bar CO oxidation conditions at room temperature for 1 h, the herringbone reconstruction is still present on most part of the terraces (see Fig. 1(b) and (c)). Additionally, islands of another structure with a height of about (0.09 ± 0.02) nm are visible on step edges as marked with black squares. An area as large as the black square (10 nm × 10 nm) is shown close up in Fig. 1(d). Averaging over tens of STM images the size of the unit cell is determined to be (0.50 ± 0.07) nm in the longer direction and (0.37 ± 0.03) nm in the shorter direction with the standard deviations as error. Areas with visible drift as at the top of Fig. 1(d) and 4(c) were excluded when measuring the unit cell. As shown in the ESI† (Fig. S1), the same unit cell is observed when the Au(111) surface is exposed to atomic oxygen before the exposure to atmospheric oxygen pressures. Additionally, it has been observed in STM by Min *et al.* after exposing Au(111) to ozone (see Fig. 9 in ref. 40). When using atomic oxygen, the oxide can also grow on the terraces (see Fig. S1(b) in the ESI†) as opposed to only at step edges as in Fig. 1. This allows for the observation of three different orientations of the oxide unit cell as is to be expected for a rectangular structure on top of the hexagonal Au(111) substrate. For a more reliable measurement of the unit cell a LEED measurement was performed, which gives the diffraction pattern in reciprocal space shown in Fig. 2(a). Based on the real space unit cell observed in STM the simulated LEED image shown in Fig. 2(b) was constructed taking all three different orientations into account. Comparison of the numbered spots shows the agreement between measured and simulated LEED image. Note that in order to observe the unit cell in LEED a higher coverage of the gold oxide is needed, which can only be achieved on a contaminated surface. As described below, the same surface allows for the oxygen to be detected spectroscopically. Overall, these results allow for identifying the islands as a surface gold oxide.

### The role of water

**Fig. 3:**
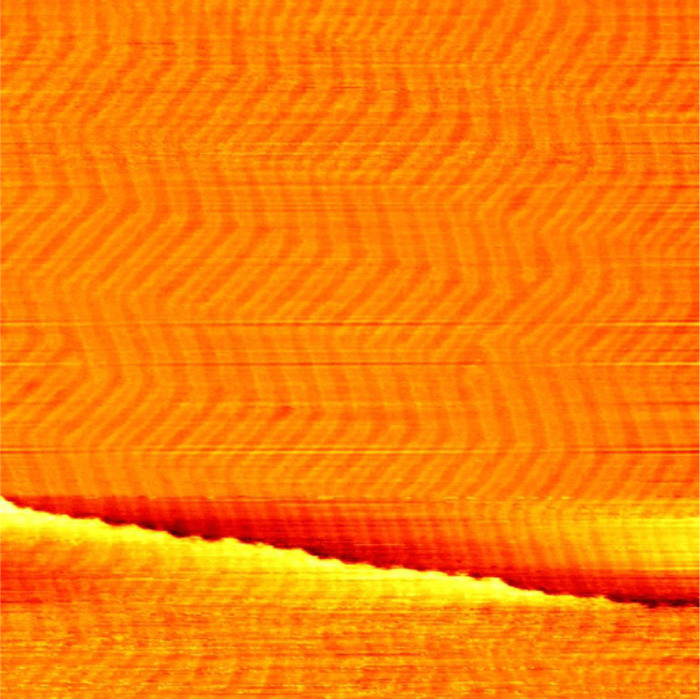
150 nm × 150 nm STM image of the as-prepared Au(111) surface after 1 h in 0.8 bar argon, containing roughly 1 mbar of water, at room temperature. The image is taken in UHV at room temperature with −1 V and 50 pA.

The theoretical dissociation barriers of O_2_ on the Au(111) terraces of 2.23 eV and on the Au(111) step edge of 1.16 eV^59^ suggest that O_2_ dissociation even on the step edge is unlikely in pure O_2_. To test whether water could deliver the necessary atomic oxygen, the as-prepared Au(111) was exposed to 0.8 bar argon for 1 h and thus the same amount of water as was present during the O_2_ exposure and in the reaction mixture. No oxide islands could be found after this water exposure and the herringbone reconstruction stayed intact as can be seen in Fig. 3. Water alone does thus not cause the formation of gold oxide, which can be understood on the hands of theoretical dissociation barriers as well: whereas the dissociation barrier of H_2_O is somewhat lower than for O_2_ on the terraces with 1.80 eV,^60^ it is slightly higher than for O_2_ at the step edges with 1.33 eV.^61^

However, Liu *et al.*^62^ have shown that the presence of water reduces the dissociation barrier of oxygen on the steps of Au(111). Depending on the amount of water the barrier can be as low as 0.15 eV. Given that our gas mixtures contain on the order of 1 mbar of water, it is probable that this water-assisted dissociation of molecular oxygen on the Au(111) steps supplies the first atomic oxygen from which the gold oxide can start growing. However, only one island per 320 nm × 320 nm is present on average after 1 h of oxygen exposure showing that this is still a rare event. Once an island of gold oxide exists, it is believed to be able to dissociate more O_2_ from the gas phase^40,63^ allowing the islands to keep growing away from the step. Comparing several images of the measurements in Fig. 1(b) and (c), the number and size of the gold oxide islands are comparable after exposure to oxygen and reaction conditions. This indicates that both the rate of initial dissociation as well as the subsequent growth of the oxide islands do not strongly depend on the exact oxygen partial pressure and gas composition at the conditions studied here.

### Sensitivity to the cleanliness of the substrate

**Fig. 4:**
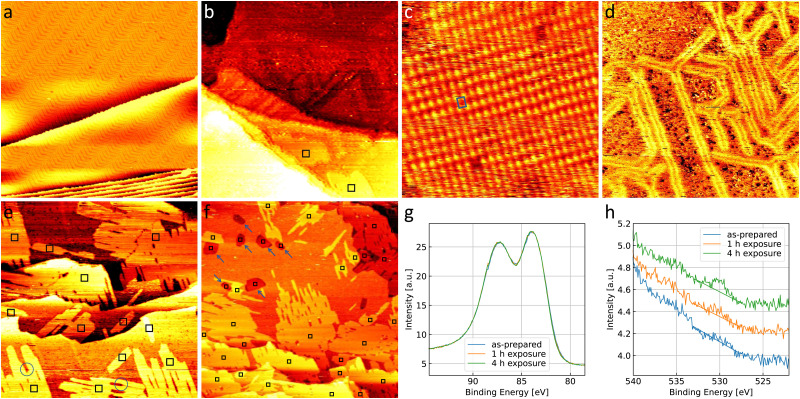
300 nm × 300 nm STM images of contaminated Au(111) (a) as-prepared and (b) after 1 h in 0.8 bar O_2_. (c) 10 nm × 10 nm image of a representative area of gold oxide with the unit cell marked in blue and (d) 120 nm × 120 nm STM image of a different terrace of the same surface. (e) 300 nm × 300 nm STM image and (f) larger 600 nm × 600 nm overview STM image of contaminated Au(111) after (e) 1 h and (f) 4 h in 1 bar of 4O_2_ + 1CO, respectively. Black squares mark islands of gold oxide, blue circles mark a certain type of contaminants that blocks the growth of islands, and blue arrows point to new step edges formed on the terrace. The images are taken in UHV at room temperature with (a) −2 V, (b) −1.5 V, (c) −1 V, (d) +1.5 V, (e) −1.5 V, and (f) −1 V, and 50 pA. (g) Au 4f and (h) O 1s spectra of the surfaces in (a), (e), and (f). All spectra are scaled with respect to the area under the Au 4f peak. The oxygen spectra are additionally shifted along the intensity axis for better visibility. Note that the oxygen peak overlaps with the onset of the Au 4p_3/2_ peak towards higher binding energies. A linear background is drawn (details see Methods section).

Fig. 4(a) shows the Au(111) substrate in a contaminated state, which is present when a newly purchased single crystal has been submitted to less cleaning cycles than needed to fully clean the crystal (as in Fig. 1(a)). Bright spots on elbows of the herringbone reconstruction as well as darker spots in between the reconstruction lines are visible on the contaminated gold. Carbon as well as other metals are typical candidates for these contaminants. However, the amount present on the surface is not sufficient to allow for a spectroscopic characterization with the methods available in the setup used here. Additionally, this means that the amount of contamination cannot be quantified precisely and repeated preparation of this surface will lead to deviations in the amount and nature of contaminants present. However, general trends can be observed compared to the clean gold when exposing a contaminated surface to the same gas environments.

Fig. 4(b) shows that the exposure to 0.8 bar O_2_ leads to a comparable number and size of gold oxide islands as on the clean gold in Fig. 1(b). Atomic resolution images show the same gold oxide unit cell (see the example given in Fig. 4(c)) as observed on the clean gold (see Fig. 1(d)). However, most of the terrace does not show the herringbone reconstruction anymore. This can be seen more clearly in Fig. 4(d). Areas which appear amorphous (meaning that no atomic resolution could be achieved) are separated by single lines of herringbone reconstruction. This suggests a mobility of the surface gold atoms during O_2_ exposure. A restructuring of Au(111) can be expected from literature under exposure to atomic oxygen (or ozone)^64,65^ as well as in O_2_ at elevated temperatures.^66–68^ As the amount of gold oxide is comparable to the case of clean gold, the presence of significantly more atomic oxygen is unlikely. It is more probable that the contaminants promote the restructuring by O_2_ similar to the effect of an elevated temperature.

After exposure to 4O_2_ + 1CO (Fig. 4(e)), the number of gold oxide islands is higher than on clean gold and a few smaller oxide islands which are not connected to a step edge are observed. Fig. 4(f) shows a larger overview image of the contaminated surface after a longer exposure of, in total, 4 h in 4O_2_ + 1CO. Whereas the coverage close to the step edges (lower half and upper right corner of the image) appears larger than after the shorter exposure (compare to Fig. 4(e)), the larger terrace in the upper left part of the image shows significant bare parts. This confirms the relevance of the step edges for the formation of most of the oxide islands, which in turn leads to a significant inhomogeneity complicating quantitative analysis of oxide coverages in the STM. Therefore, spectroscopy is used to probe a larger surface area in the next section.

Overall, this suggests that the contaminants form sites on the step edges (as well as some on the terraces) where oxygen can dissociate aided by the water (and possibly the CO) more readily than on clean gold. It is possible that these dissociation sites are the contaminants themselves, low-coordinated Au atoms caused by the contaminant, or a combination of both. Possible contaminants on Au(111) could be other metals like Pd and Pt, which are known to dissociate O_2_ directly.^31^ However, the CO would also adsorb more readily on other metals, like for example on single crystals of Pd and Pt compared to Au(111),^69^ and the presence of CO is known to hamper the O_2_ dissociation on these surfaces.^70,71^ Therefore, the presence of these metals could not fully explain the behavior observed here in the reaction mixture compared to O_2_. Lastly, silver is the most likely bulk contaminant in the gold single crystal according to the analysis provided by the supplier. Although Ag(111) is inert for O_2_ dissociation, low-coordinated Ag sites might easily split O_2_.^31^ At the same time the adsorption of CO on Ag is expected to be even weaker than on Au^69^ such that it cannot block sites for the O_2_ dissociation, which would therefore agree better with our results. Overall, as a spectroscopic identification is not possible, the nature of the contaminants remains speculative.

We do not only observe more gold oxide islands, but a single island can also grow larger than on clean gold during the same exposure time. As we identify the O_2_ dissociation on already oxidized gold as responsible for this above, we have to conclude here that the dissociation promoted by contaminants is more efficient than the self-catalyzed growth or that the latter is promoted by the contaminants as well.

In Fig. 4(e) blue circles mark positions where it can be seen that contaminants, possibly of a different nature, are able to block the growth of the oxide islands as well, which leads to more irregular shapes. As a common contaminant that is known to form pinning sites on Au(111) and does not promote O_2_ dissociation, it is likely that this contaminant is carbon.

Apart from the gold oxide islands, the terraces of the gold substrate are influenced as well. Similar to after oxygen exposure, a lifting of the herringbone reconstruction is observed with only a few lines left after exposure to the reaction mixture. After the longer exposure one can even find additional step edges forming one layer thick protrusions on the terraces, most of which contain a gold oxide island, as marked with blue arrows in Fig. 4(f). As this restructuring generally suggests that the surface atoms of gold are mobile during the exposure, it is possible that it aids in the formation of sites of low-coordinated gold atoms and/or contaminants where stronger O_2_ dissociation takes place. The more complete lifting of the herringbone reconstruction in the reaction mixture compared to O_2_ could be expected due to the presence of CO. Although UHV and theoretical studies suggest that it only adsorbs on low-coordinated sites of Au at room temperature,^72,73^ CO has been shown to induce mobility of surface gold atoms and lift the herringbone reconstruction on the terraces of Au(111) at sufficiently high pressures.^74,75^

### Oxygen coverage

The higher coverage with gold oxide on the contaminated surface allows for a spectroscopic investigation shown in Fig. 4(g) and (h). As explained in the introduction, a balance between making an oxide visible and limiting the interaction of the XPS measurement with the oxide needs to be found. The lab source used here allows to observe the oxygen peak when 40 consecutive scans are averaged, but leads to low energy resolution and a low signal-to-noise ratio. In the STM, the amount of surface oxide before and after the XPS measurement is comparable, therefore not suggesting a beam effect.

As no change in the gold spectra in Fig. 4(g) is observed we cannot detect a non-metallic oxidation state of the gold. This could however be missed due to the low energy resolution of the setup. The presence of weakly adsorbed oxygen contributing to the O 1s signal is unlikely as the surface is in UHV for a time on the order of tens of hours before and during the XPS measurements. Analyzing the oxygen spectra in Fig. 4(h) in detail with the procedure described in the Methods section, we roughly estimate an oxygen coverage of 9% of a monolayer after 1 h of exposure and 17% of a monolayer after 4 h of exposure. The design of the high-pressure STM^44^ is such that not the entire crystal but only the center is exposed to the gases (and studied by STM), whereas the XPS probes a larger area. With an estimate that this area is roughly three times the area exposed to the gases, the coverage can be converted to 27% and 51% expected in the area exposed to the gases for 1 h and 4 h, respectively. This is in qualitative agreement with the coverage observed in the STM (see Fig. 4(e) and (f)).

These results suggest that the growth in the first hour of exposure is significantly faster than in the following three hours. This can be understood from the inhomogeneity seen after the long exposure: in the high coverage area close to the step edges, other islands (possibly with different orientations), contaminants, as well as the step edges themselves will block the growth of existing islands after certain sizes are reached. At the same time the other areas with lower step density are still free of oxide, because the start of a new island is a rare event and in most cases requires a step edge.

### Gold oxide on TiO_2_/Au(111)

**Fig. 5:**
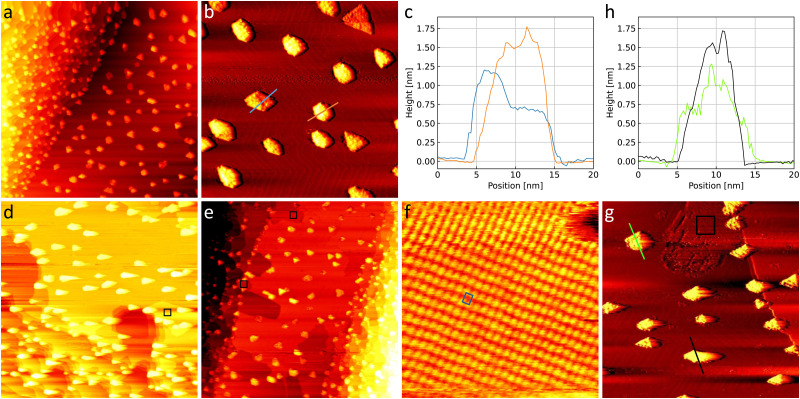
(a) 300 nm × 300 nm and (b) 110 nm × 110 nm STM images of the as-prepared TiO_2_/Au(111) with (c) corresponding height profiles. (d) 300 nm × 300 nm STM image of TiO_2_/Au(111) after 1 h in 0.8 bar O_2_ and (e) 1 h in 1 bar 4O_2_ + 1CO, respectively. (f) Representative area of a 10 nm × 10 nm black square showing the gold oxide unit cell in blue. (g) 110 nm × 110 nm STM image of a detail of the surface in (d) with corresponding height profiles in (h). In order for the particles and the herringbone reconstruction to be visible simultaneously the images in (b) and (g) were flattened and merged with their derivative in a ratio of 1 : 2 whereas the corresponding profiles in (c) and (h) are taken from the images themselves. Note that the gold oxide island marked with the black square in (g) appears darker in some areas due to an interaction of the STM tip with the titania particles after the gas exposure (details see ESI†). All images are taken in UHV at room temperature with +3 V and 50 pA.

Fig. 5(a) and (b) shows the as-prepared TiO_2_/Au(111) model catalyst. The nanoparticles are roughly between 5 nm and 15 nm wide. Triangular, hexagonal, and more elongated shapes can be recognized suggesting a crystalline structure. As can be seen in the height profiles in Fig. 5(c) particles can have a height of 0.6 nm or roughly multiples thereof with the blue height profile showing a lower and a higher side and the orange height profile showing up to 1.8 nm. Particle heights above 1.9 nm were not found in the STM. In general, the TiO_2_ nanoparticles are in agreement with those prepared by Biener *et al.*^22^ Comparable heights found by Potapenko *et al.*^76^ might suggest that the particles consist of double layers of rutile TiO_2_(100) but this could not be confirmed by atomic resolution images here.

The as-prepared TiO_2_/Au(111) surface is compared to the same surface after exposure to O_2_ and CO oxidation conditions in Fig. 5(d) and (e), respectively. Under the gas exposure, islands of gold oxide are formed at step edges as marked with black squares. The oxide was identified in zoomed-in images comparable to the example in Fig. 5(f), which shows the same unit cell as in Fig. 1(d), 4(c), and Fig. S1(a) (ESI†). The amount of gold oxide on TiO_2_/Au(111) is comparable to the amount seen when exposing only the clean Au(111) substrate (compare to Fig. 1(b) and (c)). Specifically, no oxide islands are observed on the terraces despite the presence of the TiO_2_ nanoparticles excluding that the titania has provided significant amounts of atomic oxygen to the gold surface.

Fig. 5(g) shows the nanoparticles after the gas exposure next to one island of gold oxide at a step edge. The corresponding height profiles depicted in Fig. 5(h) show that the nanoparticles still have the characteristic heights of roughly multiples of 0.6 nm. However, one might find them to have a slightly more irregular height and they clearly interact with the STM tip, which moves from left to right while recording the image in Fig. 5(g) dragging out the brighter contrast of the particles. This frequently leads to a different appearance of the gold oxide (see ESI† for details) and might lead to the particles themselves appearing more corrugated as well. Overall, this suggests that the particles likely interact in some way with the gases during the exposure. Studies on TiO_2_ single crystals suggest that dissociation of molecular oxygen is possible at oxygen vacancies in the titania at room temperature.^77^ Additionally, the dissociation is increasingly more likely with higher vacancy density.^78^ If oxygen dissociation does take place on the titania in our case, the resulting oxygen atoms might thus rather remain on the nanoparticles curing vacancies instead of spilling over to the gold substrate. Further detailed studies on the number and nature of defects on the titania nanoparticles, which are hampered here by the interaction with the STM tip and the gases, would be necessary to unambiguously exclude atomic oxygen spillover as a possible reaction mechanism on industrial catalysts.

## Conclusions

We have presented evidence for the formation of surface gold oxide on Au(111) and TiO_2_/Au(111) model catalysts under exposure to O_2_ or 4O_2_ + 1CO at atmospheric pressures and room temperature. The formation is likely enabled by water and can be strongly promoted by contaminants on the Au(111) substrate. Taking into account that under industrial conditions the same or more water is present and the gold is less pure, it is reasonable to assume that gold oxide could be formed during the reaction on a realistic catalyst.

As we do not see any influence of titania on the gold oxide formation, we cannot confirm that the transfer of atomic oxygen from titania to gold is a possible step in the CO oxidation mechanism at ambient pressures and room temperature. Assuming that our conclusion about the role of water is correct, our observations cannot corroborate that titania and water have interchangeable roles as suggested in ref. 39.

To clarify whether the gold in the surface oxide is in a non-metallic oxidation state, synchrotron near-ambient pressure XPS will be crucial.

## Author contributions

S. Wenzel: main investigation, writing. D. Boden: investigation (preparation of TiO_2_/Au(111) and LEED). I. M. N. Groot: supervision, funding acquisition.

## Data availability

Data are available from the corresponding author upon request.

## Conflicts of interest

There are no conflicts to declare.

## Supplementary Material

CP-026-D4CP00611A-s001
