# Supplementary material for: Gold oxide formation on Au(111) under CO oxidation conditions at room temperature
Source: Phys Chem Chem Phys. 2024 Aug 29;26(36):23623–30. doi: 10.1039/d4cp00611a (PMC11359969; doi:10.1039/d4cp00611a)
Supplement: CP-026-D4CP00611A-s001 [file CP-026-D4CP00611A-s001.pdf]

Supplementary Information (SI) for Physical Chemistry Chemical Physics.
This journal is © the Owner Societies 2024

Supplementary Information to "Gold Oxide Formation on Au(111) under CO Oxidation Conditions at Room Temperature"

Sabine Wenzel,*[a,b] Dajo Boden,[a] and Irene M.N. Groot[a]

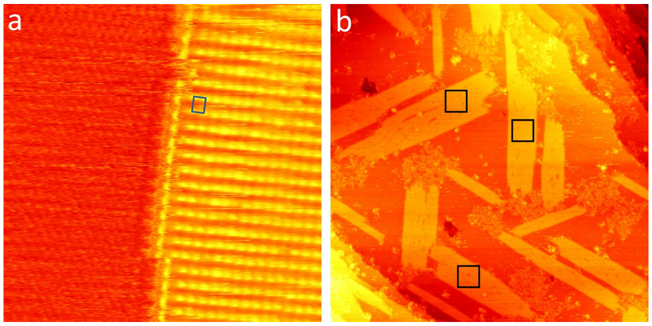

Fig. S.1 (a) 10 nm x 10 nm STM image of O/Au(111) prepared using a hot tungsten filament in oxygen background and subsequently exposed to 1 bar O_2 for several hours. The rectangular unit cell of gold oxide is marked in blue and the Au atomic rows are faintly visible on the left. (b) 140 nm x 140 nm STM image of the same surface showing the three possible orientations of gold oxide islands marked with black squares of 10 nm x 10 nm. The images are taken in UHV at room temperature with (a) +0.3 V and 140 pA, and (b) +1 V and 80 pA.

Gold Oxide Formed from Atomic Oxygen

For comparison to the surfaces studied in the main text, one gold sample was oxidized in O_2 after depositing a seed of atomic oxygen. In this case a tungsten filament facing the surface was turned on in an oxygen background on the order of $1 \cdot 10^{-5}$ mbar for 75 min before exposure to atmospheric pressures of O_2. As shown in Figure S.1(a), this leads to the formation of the same structure as described in the main text as evidenced by the same rectangular unit cell. In the larger overview in Figure S.1(b), it can be seen that the use of an atomic oxygen seed allows for the growth of gold oxide islands which are not connected to a step edge. In this case, the three possible orientations of the rectangular gold oxide unit cell on top of the hexagonal unit cell of the gold substrate can be seen.

Appearance of Gold Oxide in the Presence of Titania

Figure S.2 shows how the gold oxide can appear differently when titania is present. In order to be able to image the titania nanoparticles positive bias voltages of +3 V (corresponding to empty-state scanning) are necessary. At these conditions, especially when titania particles are in the scanned area as well, the imaging of the gold oxide can switch between appearing higher or lower than the gold substrate. The left height profile (in blue) shows the apparent height of around 0.1 nm which is also measured in all conditions without titania at other voltages between −1.5 V (filled-state scanning) to +1 V (empty-state scanning) as in the examples given in the main text. In contrast, the right height profile (in orange) shows an appearance of 0.04 nm lower than the substrate, which must be due to the electronic structure of the tip at the moment. In comparison with Figure 5(g) in the main text, which was measured in the same area with the tip moving in the opposite direction, one can notice that the gold oxide appears lower more frequently when the tip moved over a nanoparticle before imaging the gold oxide. Due to the presence of the titania nanoparticles, non-metallic material might be attached to the tip possibly creating a new apex where the electrons tunnel through and effectively decreasing the tunneling probability. This could also effect the appearance of the titania particles themselves although similar heights are measured before and after the gas exposure (see main text).

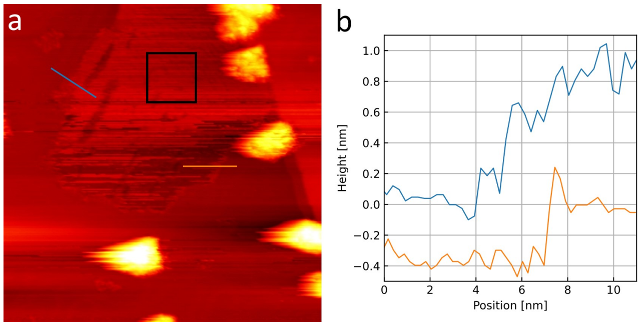

Fig. S.2 (a) 65 nm x 65 nm STM image of TiO_2/Au(111) after 1 h in 0.8 bar O_2 with corresponding height profiles. The image shows the same area as the image in Figure 5(g) in the main text but was measured with the STM tip moving from right to left instead of left to right. A black square marks a gold oxide island. The image is taken in UHV at room temperature with +3 V and 50 pA.

Author Contributions

S. Wenzel: main investigation, writing
D. Boden: investigation (preparation of TiO_2/Au(111) and LEED)
I.M.N. Groot: supervision, funding acquisition

Conflicts of interest

There are no conflicts to declare.

Acknowledgements

We thank Rik V. Mom and Mahesh K. Prabhu for fruitful discussion. This work was supported by the Dutch Research Council (NWO), BASF Nederland B.V., SABIC Global Technologies B.V., and Sasol Technology (Pty) Ltd. [grant number 731.015.604].

[a] *Leiden Institute of Chemistry, Einsteinweg 55, 2333 CC Leiden, The Netherlands*
[b] *Current Address: Department of Chemistry, University of Marburg, 35032 Marburg, Germany. E-mail: sabine.wenzel@uni-marburg.de*
